# Genetic ancestry, admixture, and population structure in rural Dominica

**DOI:** 10.1371/journal.pone.0258735

**Published:** 2021-11-03

**Authors:** Monica H. Keith, Mark V. Flinn, Harly J. Durbin, Troy N. Rowan, Gregory E. Blomquist, Kristen H. Taylor, Jeremy F. Taylor, Jared E. Decker

**Affiliations:** 1 Department of Anthropology, University of Missouri, Columbia, Missouri, United States of America; 2 Division of Animal Sciences, University of Missouri, Columbia, Missouri, United States of America; 3 Genomics Center for the Advancement of Agriculture, University of Tennessee Institute for Agriculture, Knoxville, Tennessee, United States of America; 4 Department of Anatomy and Pathological Sciences, University of Missouri, Columbia, Missouri, United States of America; Universitat Pompeu Fabra, SPAIN

## Abstract

The Caribbean is a genetically diverse region with heterogeneous admixture compositions influenced by local island ecologies, migrations, colonial conflicts, and demographic histories. The Commonwealth of Dominica is a mountainous island in the Lesser Antilles historically known to harbor communities with unique patterns of migration, mixture, and isolation. This community-based population genetic study adds biological evidence to inform post-colonial narrative histories in a Dominican horticultural village. High density single nucleotide polymorphism data paired with a previously compiled genealogy provide the first genome-wide insights on genetic ancestry and population structure in Dominica. We assessed family-based clustering, inferred global ancestry, and dated recent admixture by implementing the fastSTRUCTURE clustering algorithm, modeling graph-based migration with TreeMix, assessing patterns of linkage disequilibrium decay with ALDER, and visualizing data from Dominica with Human Genome Diversity Panel references. These analyses distinguish family-based genetic structure from variation in African, European, and indigenous Amerindian admixture proportions, and analyses of linkage disequilibrium decay estimate admixture dates 5–6 generations (~160 years) ago. African ancestry accounts for the largest mixture components, followed by European and then indigenous components; however, our global ancestry inferences are consistent with previous mitochondrial, Y chromosome, and ancestry marker data from Dominica that show uniquely higher proportions of indigenous ancestry and lower proportions of African ancestry relative to known admixture in other French- and English-speaking Caribbean islands. Our genetic results support local narratives about the community’s history and founding, which indicate that newly emancipated people settled in the steep, dense vegetation along Dominica’s eastern coast in the mid-19^th^ century. Strong genetic signals of post-colonial admixture and family-based structure highlight the localized impacts of colonial forces and island ecologies in this region, and more data from other groups are needed to more broadly inform on Dominica’s complex history and present diversity.

## Introduction

The Caribbean is a genetically diverse region where migrations, specific island ecologies, and colonial conflicts have locally shaped demographic patterns and population structures [1–4]. The Commonwealth of Dominica is a mountainous island nation in the Lesser Antilles where exceptionally steep terrain is historically known to have provided refuge for people of indigenous and African ancestries fleeing colonial violence and enslavement between the late 15^th^ and mid-19^th^ centuries [5, 6]. Mitochondrial and Y chromosome data indicate that uniquely higher proportions of indigenous Amerindian genetic lineages have survived in Dominica than among neighboring Caribbean islands, but genome-wide patterns of extant variation in Dominica have yet to be characterized [7, 8]. We assess population structure and genetic ancestry in a horticultural community on the eastern coast of Dominica using high-density single nucleotide polymorphism (SNP) and genealogical data to inform post-colonial history in this unique region with biological evidence.

Archaeological, historical, and genetic data indicate that populations across the Antilles have had complex histories and interactions over at least 6,000 years of human occupation in the Caribbean [9]. Current research suggests that Ceramic Age (500 BCE-1500 CE) populations throughout the Lesser Antilles shared a common genetic origin with a single migratory expansion from northern South America [9, 10], which may have brought the earliest human inhabitants to Dominica ~3,000 BCE [6]. Movements and interactions between different Amerindian groups across Archaic, Ceramic, Colonial, and Post-Colonial periods in the Lesser Antilles remain unresolved, and preservation of information and materials has been hindered by colonial violence and the tropical ecology. Thus far, genetic data suggest low affinity between sampled ancient and extant Caribbean groups, and many lineages appear to have been lost or redistributed as a consequence of colonial violence and displacement [11]. However, indigenous lineages survive into the present through localized admixture and among distinct ethnic communities such as Santa Rosa First Peoples in Trinidad [12] and the Kalinago Territory in Dominica [5].

Multiple Amerindian groups are known to have joined forces in Dominica against Spanish invaders following Christopher Columbus’s contact in 1493, and it is estimated that the Kalinago population declined by as much as 90% between the late 15^th^ and early 18^th^ centuries as Spanish, British, and French conquests reached the area [5]. Labor from enslaved and indentured African, European, and indigenous groups enabled a mix of French and British plantations to produce coffee and sugar in Dominica throughout the 17^th^ and 18^th^ centuries, until approximately 14,000 people were legally emancipated in 1834 [5, 6]. In 1903, the government designated 3,700 acres of land along the island’s northeastern coast as indigenous Kalinago Territory, which is currently home to approximately half of the population in St. David’s Parish (N = 6,043) [13].

Bwa Mawego is a rural horticultural community in Dominica located on the island’s steep eastern coast, south of the indigenous Kalinago Territory. This village is one of the most remote on the island and is thought to have been populated by newly emancipated people who settled in the exceptionally steep windward landscape during the 19^th^ century [14]. Challenging to traverse even on foot, Bwa Mawego lies in dense vegetation at the end of a sharply winding road along the mountainous eastern cliffside of the island. The majority of Bwa Mawego’s residents (~500) have been engaging in anthropological and psychosocial health research for the past 30 years [15–17]. Population-specific heritability estimates derived from genealogical data indicate that substantial proportions of variation observed in longitudinal health traits are explained by genetic variation [18], yet genetic variation in this region has yet to be explored in detail. Prompted by local interest in the village’s founding and ancestry, our objectives for this paper are to characterize population structure, genetic ancestry, and recent admixture in Bwa Mawego using high-density genotype data. Also related to local interest in mapping genotype-health phenotype associations, these population genetic assessments will inform our ability to effectively model population structure in subsequent medical genetic analyses. Caribbean and Latin American groups are heterogeneous in their ancestral compositions with varying degrees of admixture from indigenous Amerindian, European, and African groups [1, 3, 7]. People with recently mixed ancestries are under-represented in genetic research [19–21], and relatively isolated communities may have otherwise rare genetic variants reach detectably high frequencies, reflecting unique local histories, adaptations, and founder effects [22, 23]. As genetic data becomes increasingly informative in managing health and complex diseases, analyses of admixed genomes improve our understanding of polygenic traits, enhance trait mapping, and mitigate the lack of globally diverse representation in genetic research [24, 25].

An analysis of admixture throughout the English-speaking Caribbean that used a targeted set of ancestry informative markers found significantly more indigenous and European ancestry in Dominica relative to all other islands that had more African ancestry, indicating that patterns of genetic admixture in Dominica are unique [26]. Our samples from a localized horticultural community capture genome-wide variation in rural Dominica with a high-density SNP array [27]. Genetic research that is inclusive to people from ancestrally heterogeneous populations, such as those in the Caribbean and Latin America, requires sampling and analyses at finer scales in order to account for the complexity and diversity of specific admixtures and population structures that vary in a highly localized manner [18, 28]. Here, we analyze population structure, genetic ancestry, and admixture in a community that is both culturally and geographically defined in a unique region of the Caribbean. Our results capture well-defined genetic structure in rural Dominica, distinguishing patterns in family-based relatedness from those reflecting admixed genetic ancestry, and we effectively date recent admixture in Bwa Mawego in support of the community’s narrative history.

## Results

**Fig 1:**
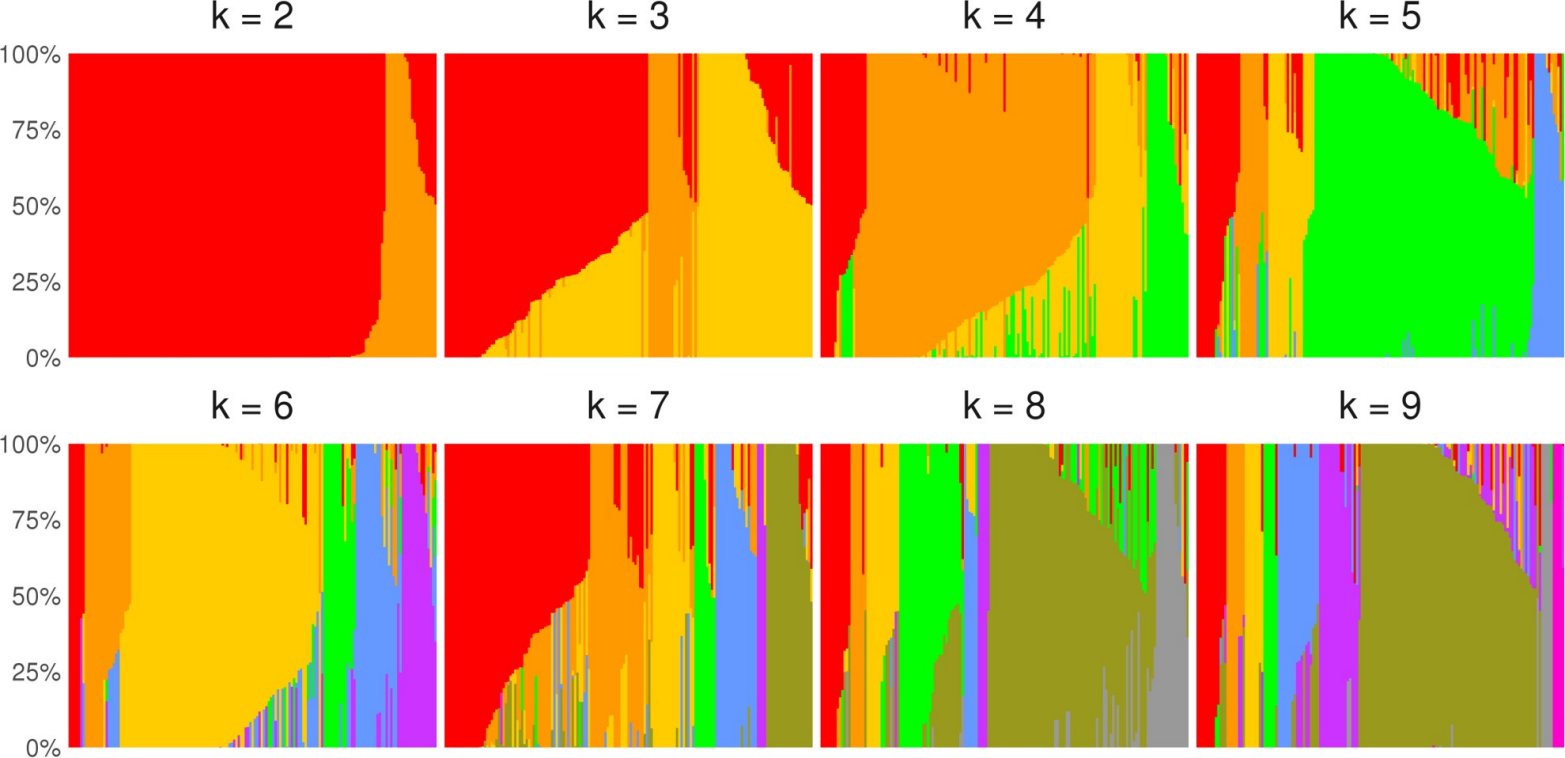
Genetic structure in Bwa Mawego. Each color represents a cluster (for K number of clusters in each model) and each bar shows individual cluster proportions ordered across panels (n = 159).

We assessed population structure in Bwa Mawego, Dominica using 468,721 SNPs genotyped in a sample of 159 people using fastSTRUCTURE [29]. The lower bound estimate of *K*_*max*_ = 4 indicates that four clusters maximize the marginal likelihood of observed genetic variation, and the upper bound estimate of *K* = 9 accounts for additional weaker population structure in Bwa Mawego (Fig 1). We utilized the *K*_*max*_ = 4 clusters in subsequent analyses, retaining each individual’s affinity to these four groups. Four Bayesian random effects models compared these cluster affinities with a previously compiled 11-generation population-wide pedigree (S1 Table in S1 File) [30], producing a heritability estimate for each cluster that indicates the proportion of cluster affinity explained by family relatedness. These models show that the four-cluster genotype structure largely reflects recently-derived family relatedness rather than more distant admixture or other potential sources of population genetic structure (S1 Fig in S1 File). Among 91 individuals with both genotype and pedigree data, pedigree-derived relatedness explains approximately 99% of red cluster affinity, 78% of orange affinity, 73% of yellow affinity, and 99% of green cluster affinity, calculated as heritability proportions from each model’s variance components (S2 Table in S1 File). Marking individuals with affinities >0.90 on the pedigree chart also shows these genetic clusters to be localized in family lineages, reflecting recent family-based structure in the community (S1 Fig in S1 File).

**Fig 2:**
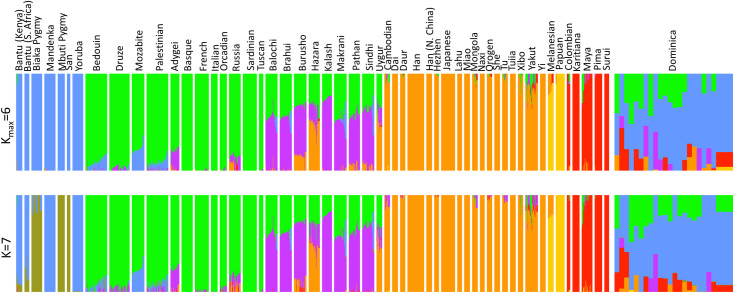
Admixture proportions in HGDP and Dominica samples. Bars show cluster proportions among 919 HDGP references and 22 unrelated Dominica samples for K_max_ = 6 (top panel) and K = 7 (bottom panel) clusters.

**Fig 3:**
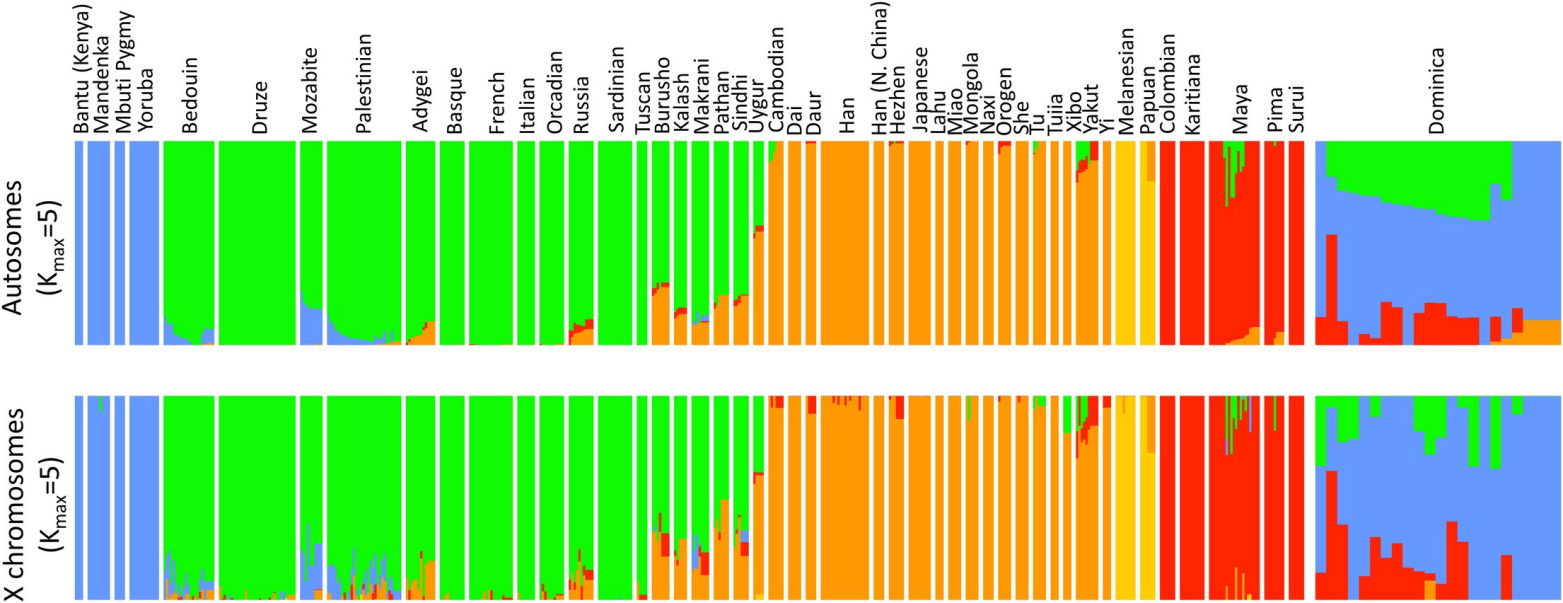
Autosomal and X chromosomal admixture proportions among females. Bars show K_max_ = 5 cluster proportions along autosomes (top panel) and X chromosomes (bottom panel) among 336 women sampled from Dominica and the HGDP.

We inferred global ancestry in Bwa Mawego using a subset of 22 individuals that excluded close relatives (r<0.025) in reference to data from 919 people representing 53 populations in the Human Genome Diversity Panel (HGDP) dataset [31]. The lower bound from fastSTRUCTURE indicates that six clusters maximize the marginal likelihood of the combined HDGP data and 22 Dominica samples, and that seven clusters maximize the amount of variation explained when accounting for additional weaker substructure (Fig 2). Bwa Mawego samples share cluster affinities with African, European, and Amerindian populations in substantial proportions, showing clear evidence of admixture from these genetically variable ancestries. *K*_*max*_ = 5 maximized the marginal likelihood of sampled variation among only females from Dominica and the HGDP. Cluster affinities show 10–13% less European ancestry on X chromosomes than autosomes among Dominican women (Fig 3).

**Fig 4:**
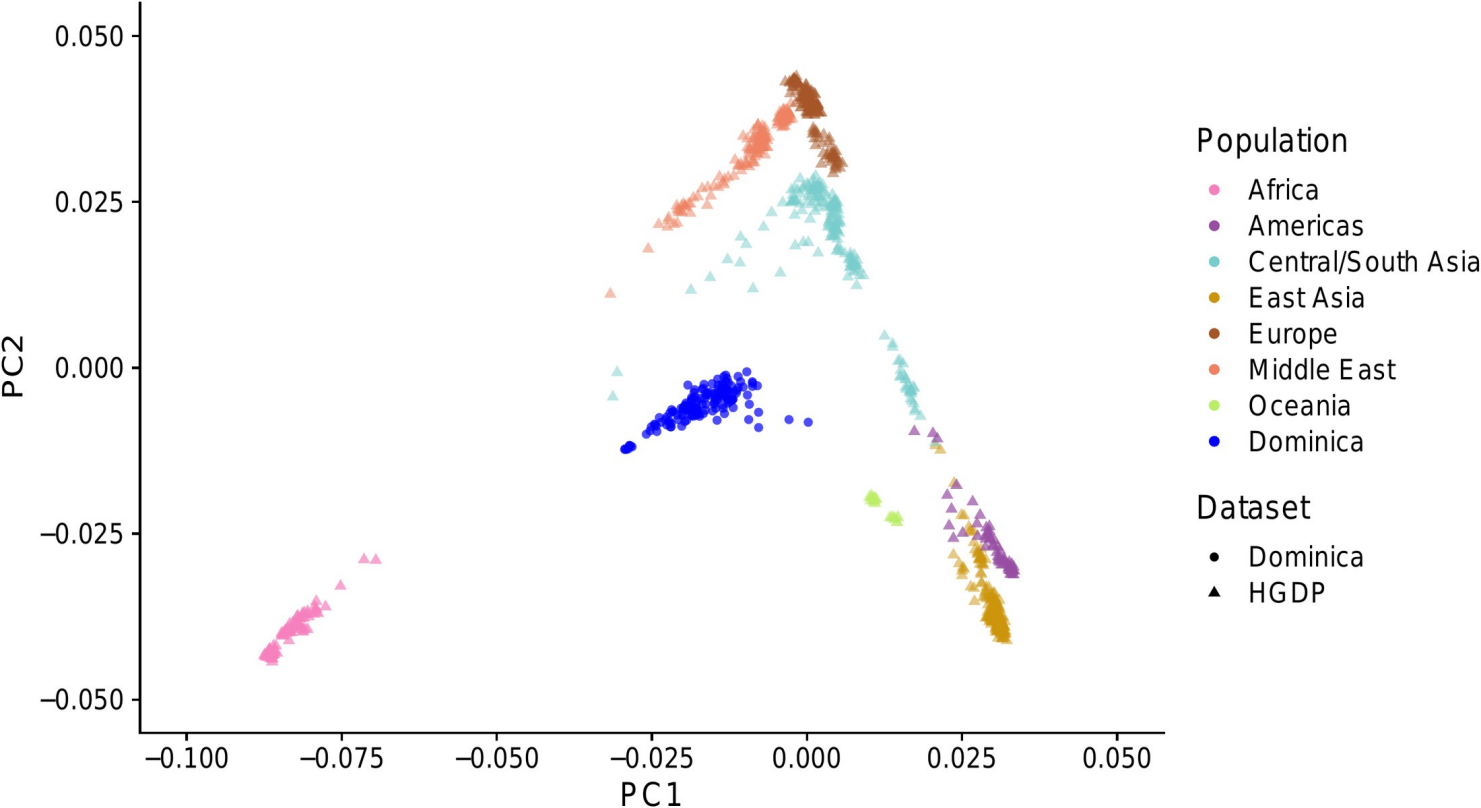
Dominica samples projected onto HGDP principal component axes. HGDP individuals are plotted as triangles, and Dominican individuals (n = 159) are plotted as closed circles in blue.

We derived the first two principal components for the HGDP reference dataset in smartpca [32] and then mapped the loadings of all 159 Dominica genotypes onto the space (Fig 4). These two principal components clearly distinguish African, European, and East Asian/Amerindian genetic clusters, and samples from Dominica form a diffuse but intermediate cluster along both axes.

Phylogenetic inferences from TreeMix [33] indicate that individuals in Bwa Mawego, Dominica share the greatest extent of their ancestry with African populations and more similarity with individuals from Yoruba and Mandenka populations than with San groups (Table 1). *f*_*3*_ statistics show the most significant negative branch lengths between Dominica and Yoruba, French, and Karitiana samples (Table 1). Strong genetic drift and founder effects can mask signals of admixture captured by this metric [34], yet we detect highly significant negative branch lengths in these data as strong evidence of admixture. We detect significant admixture from African/European and African/Amerindian source pairs but not from European/Amerindian pairs (Table 1). *f*_*4*_ admixture ratios estimate a larger contribution to genetic ancestry in Bwa Mawego from African populations than European populations, and a larger contribution from European populations than from Amerindian populations (Table 2).

**Table 1:** *f*_*3*_ statistics.

Ref A	Ref B	*f*_*3*_	Standard error	Z-score
Yoruba	French	-0.008	0.00011	-72.44
Yoruba	Orcadian	-0.008	0.00012	-69.44
Yoruba	Pima	-0.010	0.00015	-65.91
Karitiana	Yoruba	-0.011	0.00017	-64.52
Yoruba	Surui	-0.011	0.00017	-63.71
Mandenka	French	-0.007	0.00011	-63.05
Mandenka	Orcadian	-0.007	0.00012	-61.77
Mandenka	Karitiana	-0.010	0.00017	-60.67
Mandenka	Pima	-0.009	0.00016	-60.30
Mandenka	Surui	-0.010	0.00017	-58.94
San	Orcadian	-0.007	0.00016	-40.31
San	French	-0.006	0.00016	-40.04
San	Surui	-0.009	0.00024	-38.35
San	Karitiana	-0.009	0.00024	-37.56
San	Pima	-0.008	0.00022	-37.25
Surui	French	0.014	0.00030	45.67
Surui	Orcadian	0.014	0.00030	46.52
Karitiana	Orcadian	0.014	0.00028	50.77
Karitiana	French	0.014	0.00026	52.07
Orcadian	Pima	0.015	0.00027	55.44
French	Pima	0.014	0.00025	55.81
San	Yoruba	0.011	0.00014	76.83
Mandenka	San	0.011	0.00014	77.54
French	Orcadian	0.026	0.00027	97.47
Mandenka	Yoruba	0.012	0.00012	101.39
Surui	Pima	0.047	0.00043	107.80
Karitiana	Pima	0.047	0.00043	109.39
Karitiana	Surui	0.053	0.00048	110.86

Samples from Bwa Mawego, Dominica are the test population.

Negative values indicate non-phylogenetic relationships as evidence of admixture and positive values represent branch lengths from phylogenetic relationships.

**Table 2:** *f*_*4*_ ratio estimates of pairwise mixture proportions.

*f*_*4*_ ratio	Population B	Ancestry Estimate	Population C	Ancestry Estimate
(San,Basque;Yoruba,Dominica)/	French	0.370	Yoruba	0.630
(San,Basque;Yoruba,French)				
(San,Basque;Yoruba,Dominica)/	Orcadian	0.370	Yoruba	0.630
(San,Basque;Yoruba,Orcadian)				
(San,Surui;Yoruba,Dominica)/	Karitiana	0.261	Yoruba	0.739
(San,Surui;Karitiana,Yoruba)				
(San,Karitiana;Yoruba,Dominica/	Surui	0.260	Yoruba	0.740
(San,Karitiana;Yoruba,Surui)				

We ran two-reference admixture models in ALDER [35] for a subset of African, European, and Amerindian HGDP populations in relation to all 159 samples from Dominica and report date estimates from the reference pairs with significant admixture linkage disequilibrium (LD) (Table 3). Correlated background LD begins to significantly decay beginning at lengths of approximately 1.00 centimorgan. However, when Pima and Kenya Bantu were used as reference populations, background LD persisted for more than 2 centimorgans and these populations were excluded from admixture LD curve fitting. Assuming a human generation length of 29 years [36], one- and two-reference weighted LD curves indicate that the initial community admixture occurred approximately 160 years ago in rural Dominica, with slightly more recent date estimates from European and Amerindian admixtures (Table 3). Mixture proportions from single-reference models in ALDER support our *f*_*4*_ admixture ratio results (Table 2), indicating that at least 40% of the genetic ancestry in Bwa Mawego is African, more than 20% is European, and more than 6% is shared with indigenous Amerindian groups captured by the HGDP data (Table 4) [31].

**Table 3:** ALDER two-reference admixture dates.

Reference 1	Reference 2	Minimum d (cM)	Weighted LD amplitude	Date estimate	Z-score
Mandenka	Orcadian	1.2	0.00086 ± 0.00003	5.89 ± 0.22	27.15
Bantu South Africa	Orcadian	1.3	0.00088 ± 0.00003	5.89 ± 0.22	27.10
Yoruba	Orcadian	1.6	0.00089 ± 0.00003	5.81 ± 0.22	26.75
Mandenka	Tuscan	1.2	0.00080 ± 0.00003	5.84 ± 0.23	25.70
Mandenka	French	1.8	0.00083 ± 0.00003	5.81 ± 0.23	25.66
Yoruba	French	1.8	0.00088 ± 0.00003	5.82 ± 0.23	25.45
Bantu South Africa	French	1.8	0.00085 ± 0.00003	5.84 ± 0.23	25.45
Bantu South Africa	Tuscan	1.3	0.00083 ± 0.00003	5.85 ± 0.23	25.23
Yoruba	Tuscan	1.6	0.00084 ± 0.0003	5.76 ± 0.23	24.85
Mandenka	Karitiana	1.2	0.00124 ± 0.00004	5.49 ± 0.25	21.89
Yoruba	Karitiana	1.6	0.00128 ± 0.00005	5.44 ± 0.25	21.43
Bantu South Africa	Karitiana	1.3	0.00126 ± 0.00005	5.49 ± 0.26	21.35
Mandenka	Surui	1.2	0.00124 ± 0.00004	5.47 ± 0.26	21.32
Yoruba	Surui	1.6	0.00128 ± 0.00005	5.41 ± 0.26	21.14
Bantu South Africa	Surui	1.3	0.00126 ± 0.00005	5.47 ± 0.26	20.80
Surui	Orcadian	1.0	0.00035 ± 0.00002	5.40 ± 0.30	17.92
Karitiana	Orcadian	1.1	0.00033 ± 0.00001	5.39 ± 0.32	16.64
French	Surui	1.8	0.00035 ± 0.00002	5.26 ± 0.33	16.00
Tuscan	Surui	1.0	0.00036 ± 0.00001	5.41 ± 0.34	15.77
French	Karitiana	1.8	0.00033 ± 0.00001	5.26 ± 0.34	15.29
Tuscan	Karitiana	1.1	0.00035 ± 0.00001	5.39 ± 0.36	15.06

Pairs of HGDP reference populations with significant associations in LD decay curves among Dominica samples. Curve fitting was started at genetic distances greater than minimum d to avoid the effects of shared background LD. Date estimates and errors are in generations. LD was not significant if both reference populations were from the same continent (e.g. Reference 1 Orcadian, Reference 2 French). Z-scores indicate significance of LD curve associations and account for the stability of their decay parameters.

**Table 4:** ALDER one-reference admixture dates and mixture proportions.

Reference population	d (cM)	Weighted LD amplitude	Date estimate	Z-score	Mixture %
Yoruba	1.40	0.00023 ± 0.00001	5.42 ± 0.25	21.57	40.7 ± 0.8
Mandenka	1.00	0.00021 ± 0.00001	5.43 ± 0.28	19.35	34.5 ± 0.8
French	1.60	0.00033 ± 0.00001	6.32 ± 0.25	24.84	20.4 ± 0.5
Orcadian	0.80	0.00034 ± 0.00001	6.42 ± 0.26	25.09	19.4 ± 0.4
Karitiana	0.90	0.00070 ± 0.00001	5.62 ± 0.34	16.63	6.7 ± 0.3

Date estimates and errors are in generations, and mixture proportions are lower bound estimates. Genetic distances (d) estimate the distance to which LD among Dominica samples correlates with LD in each reference population; this is the distance at which decay curve-fitting was initiated.

## Discussion

We detected clear signals of admixture in rural Dominica approximately 160 years ago between African, European, and indigenous Amerindian ancestries, informing post-colonial history in this unique region of the Caribbean with genome-wide SNP data (Fig 2, Tables 1–4). The dating of this admixture estimate in the mid-19th century closely follows emancipation in Dominica in 1834, supporting oral accounts that communities in this area were formed by newly emancipated people seeking sustained refuge along the island’s steep eastern coast [6, 14]. Although the largest proportion of extant genetic variation in Bwa Mawego, Dominica is associated with African reference populations, followed by European and Amerindian mixture proportions (Tables 2 and 4), we detected significantly more Amerindian admixture in this area than has been identified elsewhere in the Lesser Antilles [7, 8, 12]. This reflects the locally variable impacts of colonialism throughout the Caribbean that continue to impact populations into the present.

The current population of Bwa Mawego has primarily African ancestry, more than 20% recent European ancestry, and more than 6% indigenous Amerindian genetic ancestry (Fig 2, Table 4). Bwa Mawego is geographically less than ten kilometers away from the indigenous Kalinago Territory, but we expect very localized cultural and geographic boundaries to limit gene flow in this region. The mixture proportion estimates we derived from ALDER are lower bounds, and our ability to detect indigenous ancestry in these admixed genotypes also depends on how similar surviving lineages in the Lesser Antilles are to those sampled among the HGDP Amerindian reference groups, which are proxies for ancestral populations.

Consistent with other admixture analyses across the Americas and Caribbean, patterns of African genetic ancestry in Bwa Mawego most closely resemble those among Yoruban samples in the HGDP (Tables 1 and 4), reflecting a west African origin for many lineages displaced to this region during the latter half of the transatlantic slave trade [3, 37]. We identified significant admixture LD between samples from Dominica and Yoruba, Mandenka, and Bantu South Africa (Table 3) as well as long (>2 cM) background LD with Bantu Kenya. We also detected admixture LD with indigenous Amazonian Karitiana and Surui groups; however, we did not access admixture LD between central American Pima and Dominican samples due to background LD. Although the largest ancestral genetic component (between 40–74%) in Bwa Mawego is African (Tables 2 and 4), these estimates are lower than African mixture components in other Caribbean populations and are consistent with another ancestry analysis that distinguished relatively lower African and higher Amerindian mixture proportions as unique to Dominica across the English-speaking Caribbean [26]. As expected based on Dominica’s history as a French (1715–1763) and then English (1763–1978) colony [6], European mixture components in Dominica most closely resemble French and Orcadian samples in the HGDP (Tables 2 and 4).

Some admixture in Bwa Mawego appears to be sex-biased (Fig 3). We identified 10–13% less European ancestry among X chromosomes compared to autosomes of sampled women, suggesting that there was a higher proportion of European male than female ancestry among the community’s founders. This is consistent with historical accounts and genetic data that show asymmetrical admixture reflecting colonial power imbalances across the Caribbean [8, 28], and there is prior evidence for relatively higher proportions of non-African male admixture in Dominica based on Y chromosome short tandem repeats [7]. However, we interpret our X chromosome estimates with caution given the small number of generations (5–6) since initial admixture in this community (Tables 3 and 4). Mixture fractions oscillate between males and females for up to 5–10 generations as they approach their equilibrium proportions in admixed populations due to the lack of non-pseudoautosomal recombination along X chromosomes in males [38].

Prior evidence suggests that Dominica has less genetic diversity than other Caribbean populations as a result of founder effects, which could potentially obscure demographic signals [7]. However, we were able to detect clear and distinct signals of admixture and family-based structure in Bwa Mawego (Figs 1 and 2, Tables 3 and 4). We identified four population clusters that reflect recent relatedness and family-based similarity (Fig 1 and S1 Fig in S1 File), and this genotypic family structure corroborates the previously gathered genealogical data from this community [30]. Previous interviews and the population-wide pedigree informed by multiple generations of community members indicate that Bwa Mawego was founded by four main families among whom several key marriages established the village [30]. Although the precise timing of community origin is unknown from oral or written accounts, historic maps indicate that the land was settled sometime between 1787–1840 [14], and our mid-19th century admixture date estimates align closely with this timeframe. The results from this community-based study address local interests in the village’s founding and ancestry as well as inform our ability to account for family- and ancestry-based genetic structure in subsequent analyses that will pair these SNP data with longitudinal health phenotypes.

While we identify clear admixture signals in Bwa Mawego, Dominica, admixture compositions vary among individuals and do not cluster neatly according to the four-group structure that is specific to this rural community (Figs 1 and 2). Unique genetic ancestry and haplotype structure in combination with longitudinal health data in this localized horticultural population may present unique opportunities to characterize biologically significant genetic variants through admixture mapping and other analyses that utilize population structure to inform gene-trait, gene-gene, and gene-environment associations [39]. Additionally, the shared local ecology in this population renders environmental factors less heterogeneous relative to the degree of potential confounding variation among participants sampled in most genotype-phenotype studies [40]. The combination of relatively low environmental heterogeneity, diverse admixture compositions, and clearly defined population structure indicates that this culturally and geographically defined community in Dominica holds unique potential for future admixture mapping, epigenetic exploration, and other association analyses.

Dominica’s unique admixture composition and relatively high proportion of indigenous Amerindian genetic ancestry [26] highlight the locally variable impacts of colonial forces and specific ecologies in the Caribbean. This is further emphasized by the near absence of Amerindian ancestry found among the Guadeloupe archipelago neighboring the island to the north [8]. While such absences promote narratives of indigenous “extinction” in the Caribbean, our findings from rural Dominica demonstrate that, as has been found among other living groups in Cuba and Puerto Rico, indigenous lineages survive into the present and are highly variable in their distributions [3]. Notably, samples across all fifteen provinces of Cuba indicate that admixture patterns are highly localized with larger proportions of Amerindian ancestry along the eastern coast [28]. Our findings along Dominica’s eastern coast add biological support to local narrative histories of post-colonial settlement in this area but are limited to a single community, and more data is needed from other groups to more broadly inform on the island’s history and present diversity. In this study, we detect strong signals of mid-19th century admixture in Bwa Mawego following Dominica’s emancipation. Given the Amerindian lineages surviving in Dominica, more sampling across the island could yield insights into pre-colonial Amerindian history in the Caribbean that remains unresolved [10, 11] in addition to supporting local knowledge of colonial ramifications, displacements, and post-colonial narratives.

## Materials and methods

We extracted DNA from buccal swabs to produce genotype data from 160 people in Bwa Mawego, Dominica. These data were collected during July-August 2017 following research approvals from both the University of Missouri Institutional Review Board (Project #2003854) and the local Village Council in Dominica. All participants gave written informed consent prior to any data collection, and parental consent was obtained for participants under the age of 18. Informed by the previously compiled pedigree [30] and longitudinal familiarity with the community, we collected samples across all known major family lineages for this study to create as representative a dataset as possible. These SNP data will be paired with longitudinal phenotype data in subsequent analyses, and we sampled broadly across relatives given the statistical limitations of a small population size and inherent relatedness among community members.

Buccal swabs were stabilized at room temperature using Dri-Capsules [41] during data collection, and samples were extracted with the Buccal-Prep Plus DNA Isolation Kit [42] and purified with the MinElute PCR Purification Kit [43]. The 160 samples were genotyped for 960,923 SNPs on the HumanOmniExpress BeadChip. This high-density array has genome-wide coverage and captures variants across global populations sampled in the HapMap project [31]. We filtered SNP data with PLINK v.1.90 [44] to remove markers with call rates <0.90 or Hardy-Weinberg Equilibrium p-values <1x10^-40^ and individuals with call rates <0.90. Filtering removed 1,181 SNPs due to low call rate, one SNP due to Hardy-Weinberg Equilibrium p-value, and one individual due to low call rate. We used reference genotypes from the HGDP [31] for ancestry comparisons. HGDP samples were genotyped on the Illumina 650Y array, and we filtered these data as above, also removing populations with fewer than five individuals. We merged 919 filtered HGDP reference samples with our 159 Dominican samples, and the resulting dataset contained 1,078 total individuals genotyped at 468,721 SNPs shared across panels.

We inferred population structure and admixture proportions via K-means clustering using the variational Bayesian algorithm in fastSTRUCTURE [29]. Allowing the number of clusters to vary from 1–10, we assessed genetic clustering within only Dominica genotypes as well as among the HGDP with a subset of Dominica samples. Using PLINK’s ‘—rel-cutoff’ flag, we down-sampled individuals from Dominica to exclude close relatives which produced a subsample of n = 22 individuals for which the relatedness among all pairs of individuals was r<0.025. This subset of Dominican individuals was used for clustering with the HDGP dataset in fastSTRUCTURE to infer ancestry with less confounding due to family-based structure. To visualize potential sex-biased admixture, we also ran fastSTRUCTURE to compare clustering between autosomes and the X chromosomes for 336 females from the down-sampled Dominica and HGDP datasets.

Bayesian random effects models compared genotype-based clusters with an 11-generation village-wide pedigree in order to assess family-based community structure and the extent to which genotypic clustering reflects family relatedness in this population (S1 Table in S1 File). The *K*_*max*_ from fastSTRUCTURE identified four well-defined genetic clusters in Bwa Mawego, and we modeled individual cluster affinities for these four groups as outcomes in four separate models for 91 people who were both genotyped and recorded in the previously compiled pedigree [30]. Using the MCMCglmm package in R v.3.6.3, we modeled individual identities as a random effect with the pedigree-derived kinship matrix representing the covariance among individuals to predict cluster affinity outcomes [45, 46]. This modeling framework allowed us to assess the extent to which community-wide patterns of genotypic variation reflect recent family-based relatedness in Bwa Mawego, producing variance component heritability estimates for the four genotype clusters identified with fastSTRUCTURE. Each model ran for 1,020,000 iterations with a burn-in of 20,000, and we report posterior modes, 90% credible intervals, and effective sample sizes to summarize these Bayesian posterior distributions (S2 Table in S1 File).

We used smartpca from the EIGENSOFT software suite for principal component analysis [32]. We inferred eigenvectors with only the HGDP samples and projected the principal component loadings of our 159 samples from Dominica onto the HGDP space. This enabled visualization of the Dominican genotypes against globally diverse samples while preventing our relatively large, recently admixed Caribbean sample from disproportionately influencing the principal components that more broadly reflect global genetic variation.

We used TreeMix [33] to visualize historical relationships between our Caribbean samples and HGDP references and ran the ‘threepop’ and ‘fourpop’ algorithms to calculate *f* statistics. HGDP references served as proxies for globally diverse ancestral populations from which we anticipated admixture. The *f*_*3*_ statistic tests the phylogenetic structure underlying allele frequencies among three different populations [47, 48], operating from a non-admixed null hypothesis that variation in allele frequencies follows a tree-like process of population differentiation over time with positive branch lengths. The *f*_*4*_ statistic tests the tree-like structure among four populations, allowing for one internal branch that will have a length of zero among populations with no detectable admixture [47, 48]. Using *f*_*4*_ ratio estimation, *f*_*4*_ statistics can be used to estimate ancestry contributions from two diverged populations in an admixed population of interest [49]. We estimated *f*_*4*_ admixture ratios using four different combinations of African, European, and Amerindian HGDP populations informed by initial *f*_*3*_ results. Neither *f*_*3*_ nor *f*_*4*_ statistics directly test for admixture in a fourth population derived from three divergent source populations as we expect to find in Dominica. Therefore, we interpreted these phylogenetic tests within the context of the admixture analyses.

We used ALDER to date admixture events and infer minimum mixture proportions by assessing correlations of LD decay among Dominica and HGDP reference samples [31]. Recombination events increasingly dissociate allele phase relationships each generation with a likelihood that increases with genetic distance along each chromosome. Thus, detailed evolutionary relationships can be inferred between admixed and reference populations based on the lengths of reference population haplotypes found in the admixed population under the assumption of selective neutrality [50]. We ran ALDER with pairs of HGDP reference populations, and also with individual reference populations one at a time, to estimate the timing of admixture events and the mixture proportions in rural Dominica. Together, these clustering, dimension reduction, phylogenetic, and haplotype analyses characterize genetic structure in a localized horticultural community and capture historical admixture in a unique area of the Caribbean.

## Supporting information

S1 File (PDF)
