# Supplementary material for: Genetic ancestry, admixture, and population structure in rural Dominica
Source: PLoS One. 2021 Nov 3;16(11):e0258735. doi: 10.1371/journal.pone.0258735 (PMC8565749; doi:10.1371/journal.pone.0258735)
Supplement: S1 File — (PDF) [file pone.0258735.s001.pdf]

# Supporting Information

**S1 Table. Population-wide pedigree statistics.**

| | | | |
|---|---|---|---|
| Individuals | 1455 | Maternal grandmothers | 879 |
| Maternities | 1097 | Maternal grandfathers | 842 |
| Paternities | 1062 | Paternal grandmothers | 696 |
| Full sibs | 1487 | Paternal grandfathers | 727 |
| Maternal sibs | 1992 | Maximum pedigree depth | 11 |
| Maternal half sibs | 505 | Founders | 328 |
| Paternal sibs | 1733 | Non-zero F | 114 |
| Paternal half sibs | 246 | F>0.125 | 1 |
| Mean pairwise relatedness | 0.0085 | | |

Parental, grand-parental, and sibling relationship counts include all relationships, therefore, one person may have multiple maternities, sibships, etc. F values denote inbreeding coefficients.

**S2 Table. Heritability estimates for $K_{max}$=4 `fastStructure` clusters.**

| Model | posterior mode | 90% credible interval | $n_{eff}$ |
|---|---|---|---|
| Red | 0.988 | (0.956, 0.995) | 1096 |
| Orange | 0.784 | (0.655, 0.868) | 1000 |
| Yellow | 0.729 | (0.571, 0.808) | 1290 |
| Green | 0.986 | (0.952, 0.995) | 1054 |

Posterior modes, 90% credible intervals, and effective sample sizes summarize relatedness variance components from each Bayesian random effect model's posterior distribution.

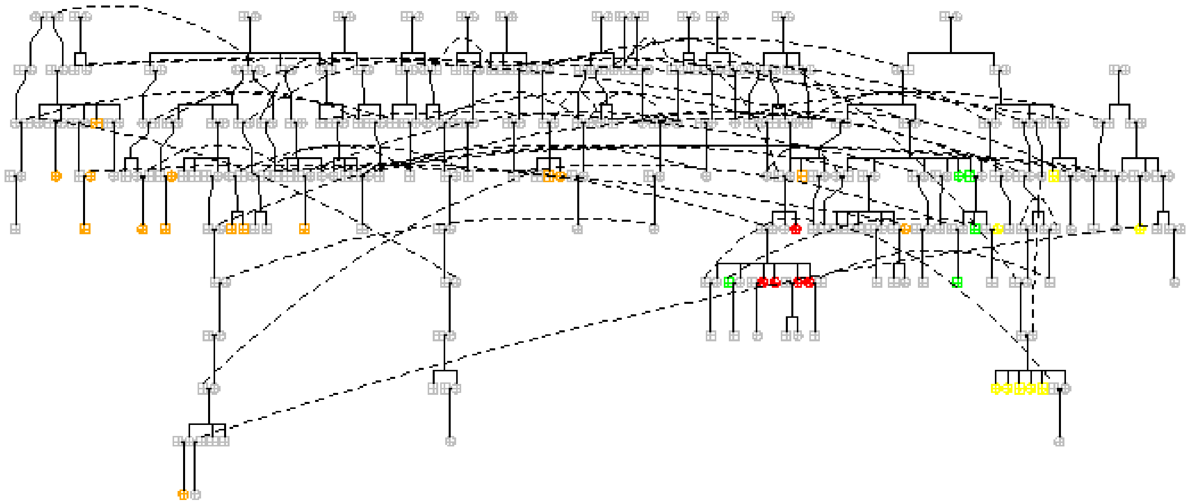

**Figure S1. Pedigree subset (n=289) that includes 91 current individuals with genotype data.**
Those with a `fastSTRUCTURE` cluster affinity >0.90 are colored accordingly ($n_{red}$=5, $n_{orange}$=14, $n_{yellow}$=8, $n_{green}$=4).
